# Crystal structure of 2,3′-bi­pyridine-2′,6′-dicarbo­nitrile

**DOI:** 10.1107/S2056989018011532

**Published:** 2018-08-21

**Authors:** Seoulgi Jung, Ki-Min Park, Jinho Kim, Youngjin Kang

**Affiliations:** a Division of Science Education & Department of Chemistry, Kangwon National University, Chuncheon 24341, Republic of Korea; b Research Institute of Natural Science, Gyeongsang National University, Jinju, 52828, Republic of Korea

**Keywords:** crystal structure, dipyridyl derivative, cyano substituent, hydrogen bonds, π–π stacking inter­actions, C≡N⋯π inter­actions

## Abstract

The title compound, C_12_H_6_N_4_, crystallizes with four independent mol­ecules (*A*, *B*, *C* and *D*) in the asymmetric unit. The dihedral angles between the two pyridine rings in each mol­ecule are 25.25 (8)° in *A*, 5.51 (9)° in *B*, 11.11 (9)° in *C* and 16.24 (8)° in *D*. In the crystal, mol­ecules *A* and *B* are linked by C—H⋯N hydrogen bonds to form layers extending parallel to the *ab* plane, while mol­ecules *C* and *D* are linked by C—H⋯N hydrogen bonds forming –*C*–*D*–*C*–*D*– chains propagating along the *b*-axis direction. The layers and the chains are stacked alternately along the *c* axis through offset π–π and C≡N⋯π [*N*-to-pyridine-centroid distance = 3.882 (2) Å] inter­actions, resulting in the formation of a supra­molecular framework.

## Abstract 2

The asymmetric unit of the title disubstituted 2,3′-bi­pyridine, contains four independent mol­ecules (namely, *A*, *B*, *C* and *D*). The conformations of the mol­ecules differ, as seen from the dihedral angles between the two pyridine rings in each mol­ecule. They vary from 5.51 (9)° for mol­ecule *B* to 25.25 (8)° for mol­ecule *A*.

## Chemical context   

Bi­pyridine ligands with the C
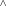
N chelating mode to transition metal ions, such as 2,3′-bi­pyridine, are considered to be strong candidates for the synthesis of blue phospho­rescent heavy transition metal complexes because of their larger triplet energy (*T*_1_) compared with phenyl­pyridine-based C
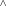
N chelating ligands (Reddy & Bejoymohandas, 2016 ▸). In particular, the triplet energy of fluorine-functionalized 2,3′-bi­pyridine (*T*_1_: 2.82 eV) is larger than that of alk­oxy-functionalized analogue, 2′,6′-dimeth­oxy-2,3′-bi­pyridine (*T*_1_: 2.70 eV) (Lee *et al.*, 2017 ▸; Kim *et al.*, 2018 ▸). Therefore, the introduction of electron-withdrawing groups into the *C*-coordinating pyridine group is highly desirable in order to develop blue phospho­rescent metal complexes. To design a suitable ligand possessing a large triplet energy is still a main issue in the organic light-emitting diodes (OLEDs) research area because developing blue phospho­rescent materials remains a problem that has not been solved so far. Although there are a number of advantages in 2,3′-bi­pyridine ligands, incorporating the substituents into the ligand framework is difficult owing to the low selectivity and reactivity of the pyridine ring (Oh *et al.*, 2013 ▸). In addition, structural examples of bi­pyridine-bearing electron-withdrawing groups are very scarce.
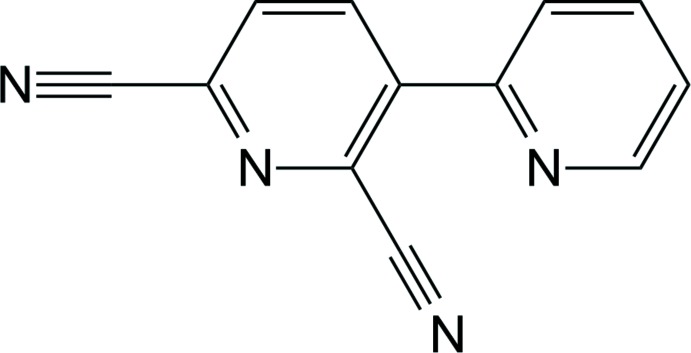


Herein, for potential applications for the development of blue phospho­rescent materials, we describe the synthesis and crystal structure of the title compound, 2,3′-bi­pyridine-2′,6′-dicarbo­nitrile.

## Structural commentary   

**Figure 1:**
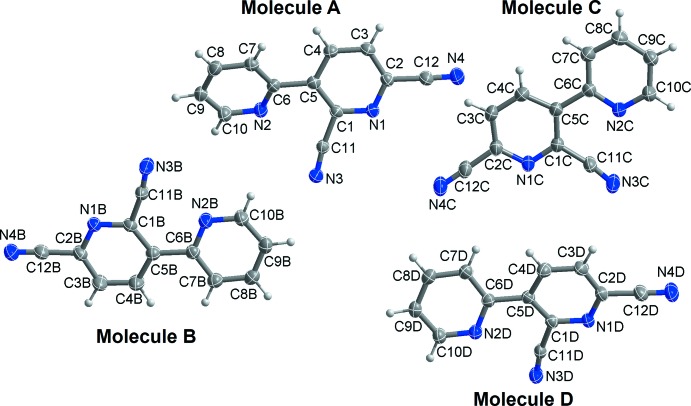
The mol­ecular structure of the four independent mol­ecules (*A*, *B*, *C* and *D*) of the title compound, with the atom-numbering scheme. Displacement ellipsoids are drawn at the 50% probability level.

**Figure 2:**
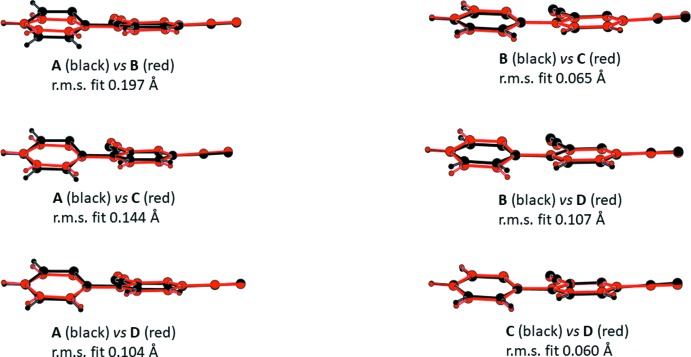
The overlay fits of the various mol­ecules in the asymmetric unit of the title compound.

As shown in Fig. 1 ▸, the asymmetric unit of the title compound contains four crystallographically independent mol­ecules (*A*, *B*, *C* and *D*). The dihedral angles between the two pyridine rings in each mol­ecule are 25.25 (8)° in *A*, 5.51 (9)° in *B*, 11.11 (9)° in *C* and 16.24 (8)° in *D*. In order to investigate the conformational similarity between the four mol­ecules, the r.m.s. overlay fits of the 16 non-H atoms of each mol­ecule were calculated using the AutoMolFit routine in *PLATON* (Spek, 2009 ▸). As shown in Fig. 2 ▸, and as expected in view of the values of the dihedral angles, the largest overlay fit of 0.197 Å is observed for mol­ecules *A* and *B*, while the smallest r.m.s. overlay fit of 0.060 Å is observed for mol­ecules *C* and *D*.

## Supra­molecular features   

**Figure 3:**
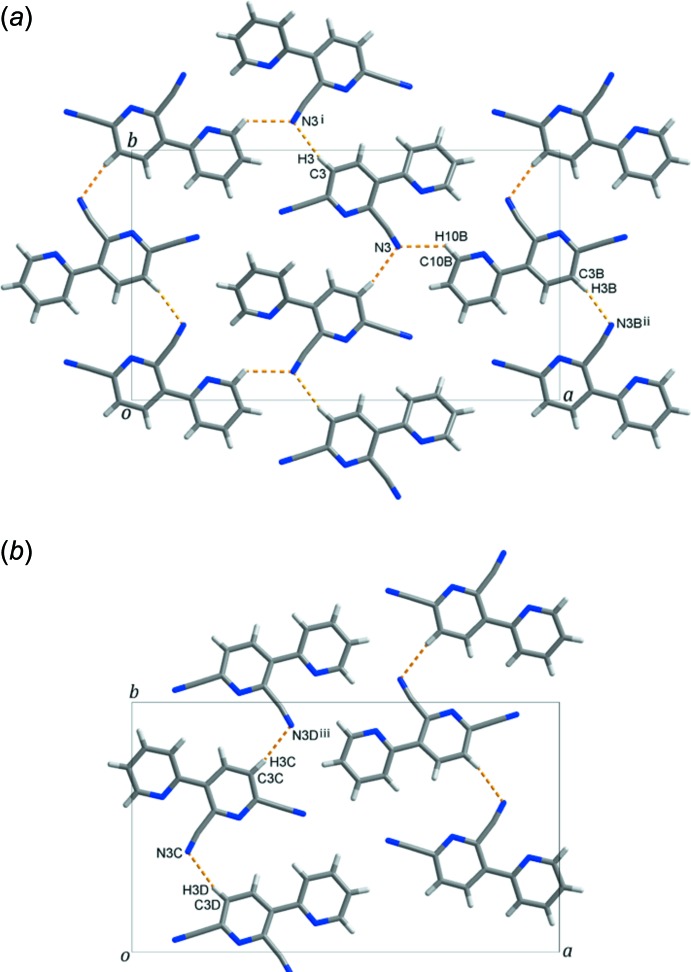
(*a*) View along the *c* axis of the layer formed by C—H⋯N hydrogen bonds between mol­ecules *A* and *B;* (*b*) view along the *c* axis of the chains formed by C—H⋯N hydrogen bonds between mol­ecules *C* and *D* [symmetry codes: (i) −*x* + 1, *y* + 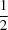, −*z* + 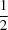; (ii) −*x* + 2, *y* − 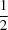, −*z* + 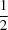; (iii) *x*, *y* + 1, *z*; colour codes: grey = carbon, blue = nitro­gen and white = hydrogen].

**Figure 4:**
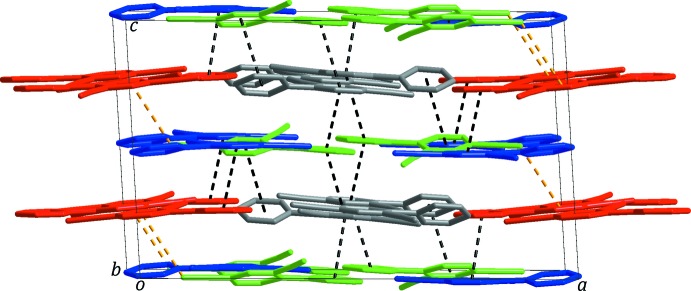
The supra­molecular framework formed *via* inter­molecular π-π stacking (black dashed lines) and C≡N⋯π (yellow dashed lines) inter­actions involving the four independent mol­ecules (colour codes: gray = mol­ecule *A*, red = mol­ecule *B*, blue = mol­ecule *C* and green = mol­ecule *D*). All H atoms have been omitted for clarity.

In the crystal, mol­ecules *A* and *B* are linked *via* C—H⋯N hydrogen bonds (C3—H3⋯N3^i^, C3*B*—H3*B*⋯N3*B* ^ii^ and C10*B*—H10*B*⋯N3, Table 1 ▸ and Fig. 3 ▸ *a*), forming layers extending parallel to the *ab* plane, while the *C* and *D* mol­ecules are connected through C—H⋯N hydrogen bonds (C3*C*—H3*C*⋯N3*D* ^iii^ and C3*D–*-H3*D*⋯N3*C*, Table 1 ▸ and Fig. 3 ▸ *b*) to from –*C*–*D*–*C*–*D*– chains propagating along the *b*-axis direction. The layers and chains stack alternately along the *c* axis, linked by inter­molecular π–π stacking inter­actions, resulting in the formation of a supra­molecular framework, as shown in Fig. 4 ▸ [*Cg*1⋯*Cg*2*D* ^i^ = 3.6741 (9) Å; *Cg*1⋯*Cg*2*D* ^iv^ = 3.6546 (9) Å; *Cg*2⋯*Cg*1*D* ^iv^ = 3.5888 (9) Å; *Cg*2*B*⋯*Cg*1*C* ^iv^ = 3.8196 (10) Å; *Cg*1 and *Cg*2 are the centroids of the N1/C1–C5 and N2/C6-C10 rings. Atoms and centroids labelled with suffixes *B*, *C* and *D* represent those of the mol­ecules *B*, *C* and *D*, respectively]. In addition, inter­molecular C≡N⋯π inter­actions between the cyano N atom of the *D* mol­ecule and the N1*B*-containing pyridine ring of mol­ecule *B* [N4*D*⋯*Cg*1*B* ^vi^ = 3.882 (2) Å; *Cg*1*B* is the centroid of the N1*B*/C1*B*–C5*B* ring; symmetry code: (vi) −*x* + 1, *y* − 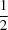, −*z* + 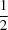], contribute to the stabilization of the framework.

## Database survey   

A search of the Cambridge Structural Database (CSD, Version 5.39, last update May 2018; Groom *et al.*, 2016 ▸) for 2′,6′-disubstituted 2,3′-bi­pyridines, gave a number of hits. The majority of them involve iridium or platinum complexes of the di­fluoro and dimeth­oxy analogues of the title compound. As explained in the *Chemical context*, such compounds, particularly blue iridium complexes of 2′,6′-di­fluoro-2,3′-bi­pyridine, have been synthesized to study their phospho­rescence (*e.g*. Lee *et al.*, 2009 ▸) and electroluminescence (*e.g*. Xu *et al.*, 2015 ▸) efficiency. As there are no reports of the crystal structures of either 2′,6′-di­fluoro-2,3′-bi­pyridine nor 2′,6′-dimeth­oxy-2,3′-bi­pyridine, it is not possible to compare their conformations with those of the four independent mol­ecules of the title compound.

## Synthesis and crystallization   

All experiments were performed under a dry N_2_ atmosphere using standard Schlenk techniques. All solvents were freshly distilled over appropriate drying reagents prior to use. All starting materials were commercially purchased and used without further purification. The ^1^H NMR spectrum was recorded on a Bruker Avance 300 MHz spectrometer. The fluorinated bi­pyridine, 2′,6′-di­fluoro-2,3′-bi­pyridine, was synthesized according to previous reports (Lee *et al.*, 2009 ▸). Then 2′,6′-di­fluoro-2,3′-bi­pyridine (2.0 g, 10.4 mmol) and sodium cyanide (1.02 g, 20.8 mmol) were dissolved in DMSO (10 ml). The reaction mixture was stirred overnight at 308 K. All the volatile components were removed under reduced pressure. The resulting mixture was poured into CH_2_Cl_2_ (20 × 3 ml), and then washed with water (3 × 50 ml) to remove any remaining sodium cyanide. Silica gel column purification with EtOAc and hexane gave a yellow powder in 60% yield. Colourless crystals suitable for X-ray crystallography analysis were obtained from a CH_2_Cl_2_/hexane solution under slow evaporation. ^1^H NMR (300 MHz, CDCl_3_, δ): 8.78 (*dd*, *J* = 3.6, 1.2 Hz, 1H), 8.40 (*d*, *J* = 8.4 Hz, 1H), 7.93–7.84 (*m*, 3H), 7.42 (*td*, *J* = 5.1, 1.5 Hz, 1H). IR(KBr, pellet): ν_CN_ = 2239 cm^−1^. Mass spectrum *m*/*z* (EI): 206 for [*M*]^+^ (calculated, 206).

## Refinement   

Crystal data, data collection and structure refinement details are summarized in Table 2 ▸. All H atoms were positioned geometrically and refined using a riding model: C—H = 0.95 Å with *U*_iso_(H) = 1.2*U*_eq_(C).

## Supplementary Material

Crystal structure: contains datablock(s) I, New_Global_Publ_Block. DOI: 10.1107/S2056989018011532/xu5937sup1.cif

Structure factors: contains datablock(s) I. DOI: 10.1107/S2056989018011532/xu5937Isup2.hkl

Supporting information file. DOI: 10.1107/S2056989018011532/xu5937Isup3.cml

CCDC reference: 1862117

Additional supporting information:  crystallographic information; 3D view; checkCIF report

## Figures and Tables

**Table 1:** Hydrogen-bond geometry (Å, °)

*D*—H⋯*A*	*D*—H	H⋯*A*	*D*⋯*A*	*D*—H⋯*A*
C3—H3⋯N3^i^	0.95	2.42	3.343 (2)	164
C3*B*—H3*B*⋯N3*B* ^ii^	0.95	2.34	3.281 (2)	169
C10*B*—H10*B*⋯N3	0.95	2.57	3.269 (2)	130
C3*C*—H3*C*⋯N3*D* ^iii^	0.95	2.46	3.379 (2)	164
C3*D*—H3*D*⋯N3*C*	0.95	2.56	3.397 (2)	145

Symmetry codes: (i) 
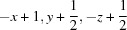
; (ii) 
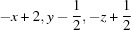
; (iii) 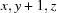.

**Table 2:** Experimental details

Crystal data	
Chemical formula	C_12_H_6_N_4_
*M*_r_	206.21
Crystal system, space group	Monoclinic, *P*2_1_/*c*
Temperature (K)	173
*a*, *b*, *c* (Å)	22.5144 (5), 13.1601 (3), 13.2652 (3)
β (°)	93.4509 (11)
*V* (Å^3^)	3923.24 (15)
*Z*	16
Radiation type	Mo *K*α
μ (mm^−1^)	0.09
Crystal size (mm)	0.40 × 0.33 × 0.29

Data collection	
Diffractometer	Bruker APEXII CCD
Absorption correction	Multi-scan (*SADABS*; Bruker, 2014 ▸)
*T*_min_, *T*_max_	0.696, 0.746
No. of measured, independent and observed [*I* > 2σ(*I*)] reflections	39169, 9671, 6997
*R*_int_	0.034
(sin θ/λ)_max_ (Å^−1^)	0.667

Refinement	
*R*[*F* ^2^ > 2σ(*F* ^2^)], *wR*(*F* ^2^), *S*	0.049, 0.140, 1.05
No. of reflections	9671
No. of parameters	578
H-atom treatment	H-atom parameters constrained
Δρ_max_, Δρ_min_ (e Å^−3^)	0.29, −0.23

Computer programs: *APEX2* and *SAINT* (Bruker, 2014 ▸), *SHELXS97* and *SHELXTL* (Sheldrick, 2008 ▸), *SHELXL2014* (Sheldrick, 2015 ▸), *DIAMOND* (Brandenburg, 2010 ▸), *PLATON* (Spek, 2009 ▸), and *publCIF* (Westrip, 2010 ▸).

## supplementary crystallographic information

### Crystal data

**Table:**

C_12_H_6_N_4_	*F*(000) = 1696
*M**_r_* = 206.21	*D*_x_ = 1.396 Mg m^−^^3^
Monoclinic, *P*2_1_/*c*	Mo *K*α radiation, λ = 0.71073 Å
*a* = 22.5144 (5) Å	Cell parameters from 9514 reflections
*b* = 13.1601 (3) Å	θ = 2.3–28.3°
*c* = 13.2652 (3) Å	µ = 0.09 mm^−^^1^
β = 93.4509 (11)°	*T* = 173 K
*V* = 3923.24 (15) Å^3^	Block, colourless
*Z* = 16	0.40 × 0.33 × 0.29 mm

### Data collection

**Table:**

Bruker APEXII CCD diffractometer	6997 reflections with *I* > 2σ(*I*)
φ and ω scans	*R*_int_ = 0.034
Absorption correction: multi-scan (SADABS; Bruker, 2014)	θ_max_ = 28.3°, θ_min_ = 0.9°
*T*_min_ = 0.696, *T*_max_ = 0.746	*h* = −29→29
39169 measured reflections	*k* = −15→17
9671 independent reflections	*l* = −17→17

### Refinement

**Table:**

Refinement on *F*^2^	Secondary atom site location: difference Fourier map
Least-squares matrix: full	Hydrogen site location: inferred from neighbouring sites
*R*[*F*^2^ > 2σ(*F*^2^)] = 0.049	H-atom parameters constrained
*wR*(*F*^2^) = 0.140	*w* = 1/[σ^2^(*F*_o_^2^) + (0.0597*P*)^2^ + 1.2183*P*] where *P* = (*F*_o_^2^ + 2*F*_c_^2^)/3
*S* = 1.05	(Δ/σ)_max_ = 0.001
9671 reflections	Δρ_max_ = 0.29 e Å^−^^3^
578 parameters	Δρ_min_ = −0.23 e Å^−^^3^
0 restraints	Extinction correction: SHELXL2014 (Sheldrick, 2015), Fc^*^=kFc[1+0.001xFc^2^λ^3^/sin(2θ)]^-1/4^
Primary atom site location: structure-invariant direct methods	Extinction coefficient: 0.00078 (18)

### Special details

**Table:**

Geometry. All esds (except the esd in the dihedral angle between two l.s. planes) are estimated using the full covariance matrix. The cell esds are taken into account individually in the estimation of esds in distances, angles and torsion angles; correlations between esds in cell parameters are only used when they are defined by crystal symmetry. An approximate (isotropic) treatment of cell esds is used for estimating esds involving l.s. planes.

### Fractional atomic coordinates and isotropic or equivalent isotropic displacement parameters (Å^2^)

**Table:**

	*x*	*y*	*z*	*U*_iso_*/*U*_eq_	
N1	0.49910 (5)	0.74057 (9)	0.26760 (10)	0.0280 (3)	
N2	0.67745 (5)	0.83351 (10)	0.29072 (11)	0.0346 (3)	
N3	0.62263 (6)	0.61415 (10)	0.24069 (12)	0.0376 (3)	
N4	0.35033 (6)	0.75276 (12)	0.29544 (15)	0.0533 (4)	
C1	0.55555 (6)	0.77161 (10)	0.26223 (11)	0.0259 (3)	
C2	0.45846 (6)	0.81336 (11)	0.27582 (12)	0.0297 (3)	
C3	0.47099 (7)	0.91693 (11)	0.27679 (13)	0.0338 (3)	
H3	0.4402	0.9658	0.2815	0.041*	
C4	0.52920 (6)	0.94626 (11)	0.27070 (12)	0.0322 (3)	
H4	0.5391	1.0165	0.2714	0.039*	
C5	0.57391 (6)	0.87387 (11)	0.26354 (11)	0.0271 (3)	
C6	0.63739 (6)	0.90300 (11)	0.25798 (12)	0.0285 (3)	
C7	0.65339 (7)	0.99737 (11)	0.22031 (12)	0.0332 (3)	
H7	0.6239	1.0453	0.1983	0.040*	
C8	0.71324 (7)	1.01972 (12)	0.21571 (13)	0.0363 (4)	
H8	0.7255	1.0838	0.1913	0.044*	
C9	0.75478 (7)	0.94792 (12)	0.24703 (13)	0.0361 (4)	
H9	0.7961	0.9608	0.2433	0.043*	
C10	0.73499 (7)	0.85668 (12)	0.28397 (14)	0.0376 (4)	
H10	0.7639	0.8075	0.3059	0.045*	
C11	0.59619 (6)	0.68697 (11)	0.25145 (12)	0.0282 (3)	
C12	0.39805 (7)	0.77851 (12)	0.28566 (14)	0.0374 (4)	
N1B	1.00098 (5)	0.66865 (10)	0.25099 (11)	0.0328 (3)	
N2B	0.82233 (6)	0.58423 (10)	0.23869 (13)	0.0426 (4)	
N3B	0.87912 (6)	0.79976 (11)	0.21706 (16)	0.0595 (5)	
N4B	1.15222 (6)	0.65194 (11)	0.28236 (15)	0.0522 (4)	
C1B	0.94394 (6)	0.63854 (11)	0.24219 (12)	0.0302 (3)	
C2B	1.04188 (7)	0.59585 (12)	0.26116 (14)	0.0368 (4)	
C3B	1.02892 (7)	0.49305 (13)	0.26150 (17)	0.0497 (5)	
H3B	1.0597	0.4437	0.2677	0.060*	
C4B	0.97019 (7)	0.46449 (13)	0.25256 (16)	0.0477 (5)	
H4B	0.9602	0.3943	0.2525	0.057*	
C5B	0.92501 (7)	0.53684 (11)	0.24356 (13)	0.0331 (3)	
C6B	0.86123 (7)	0.50763 (11)	0.23825 (12)	0.0325 (3)	
C7B	0.84293 (7)	0.40674 (12)	0.23460 (13)	0.0364 (4)	
H7B	0.8713	0.3534	0.2336	0.044*	
C8B	0.78279 (7)	0.38510 (12)	0.23251 (13)	0.0381 (4)	
H8B	0.7694	0.3166	0.2310	0.046*	
C9B	0.74263 (7)	0.46356 (13)	0.23266 (13)	0.0372 (4)	
H9B	0.7011	0.4507	0.2310	0.045*	
C10B	0.76432 (7)	0.56175 (13)	0.23535 (15)	0.0421 (4)	
H10B	0.7366	0.6162	0.2348	0.051*	
C11B	0.90390 (7)	0.72465 (12)	0.22910 (15)	0.0396 (4)	
C12B	1.10337 (7)	0.62904 (12)	0.27276 (15)	0.0408 (4)	
N1C	0.25051 (5)	0.53772 (10)	0.02073 (10)	0.0328 (3)	
N2C	0.07122 (6)	0.62043 (11)	−0.00555 (15)	0.0522 (4)	
N3C	0.13060 (6)	0.40241 (12)	0.02803 (17)	0.0616 (5)	
N4C	0.40066 (6)	0.55328 (12)	0.01479 (14)	0.0520 (4)	
C1C	0.19315 (6)	0.56632 (11)	0.02102 (12)	0.0307 (3)	
C2C	0.29079 (7)	0.61155 (12)	0.02084 (13)	0.0340 (3)	
C3C	0.27729 (7)	0.71403 (13)	0.02209 (15)	0.0429 (4)	
H3C	0.3078	0.7639	0.0238	0.051*	
C4C	0.21814 (7)	0.74172 (13)	0.02080 (15)	0.0422 (4)	
H4C	0.2077	0.8116	0.0206	0.051*	
C5C	0.17368 (7)	0.66812 (12)	0.01976 (12)	0.0325 (3)	
C6C	0.10958 (7)	0.69596 (12)	0.01452 (12)	0.0322 (3)	
C7C	0.09100 (7)	0.79532 (13)	0.02696 (15)	0.0420 (4)	
H7C	0.1192	0.8479	0.0415	0.050*	
C8C	0.03076 (7)	0.81675 (13)	0.01785 (15)	0.0445 (4)	
H8C	0.0170	0.8844	0.0257	0.053*	
C9C	−0.00892 (7)	0.73939 (13)	−0.00260 (14)	0.0402 (4)	
H9C	−0.0505	0.7522	−0.0094	0.048*	
C10C	0.01307 (7)	0.64306 (14)	−0.01301 (18)	0.0527 (5)	
H10C	−0.0145	0.5893	−0.0263	0.063*	
C11C	0.15421 (7)	0.47900 (12)	0.02419 (15)	0.0400 (4)	
C12C	0.35214 (7)	0.57814 (12)	0.01809 (14)	0.0396 (4)	
N1D	0.25087 (5)	0.03678 (9)	0.02181 (10)	0.0312 (3)	
N2D	0.42958 (5)	0.11479 (9)	−0.00983 (11)	0.0328 (3)	
N3D	0.37265 (6)	−0.09144 (10)	0.07125 (13)	0.0432 (4)	
N4D	0.10036 (6)	0.04871 (12)	0.00311 (15)	0.0545 (5)	
C1D	0.30810 (6)	0.06558 (11)	0.02102 (11)	0.0274 (3)	
C2D	0.21010 (6)	0.10823 (12)	−0.00026 (12)	0.0325 (3)	
C3D	0.22322 (7)	0.20789 (12)	−0.02331 (14)	0.0383 (4)	
H3D	0.1925	0.2561	−0.0382	0.046*	
C4D	0.28232 (7)	0.23508 (12)	−0.02402 (13)	0.0360 (4)	
H4D	0.2926	0.3030	−0.0398	0.043*	
C5D	0.32714 (6)	0.16403 (11)	−0.00185 (11)	0.0281 (3)	
C6D	0.39103 (6)	0.19201 (11)	−0.00522 (11)	0.0274 (3)	
C7D	0.40905 (7)	0.29331 (11)	−0.00522 (12)	0.0312 (3)	
H7D	0.3808	0.3464	−0.0003	0.037*	
C8D	0.46879 (7)	0.31533 (12)	−0.01251 (12)	0.0336 (3)	
H8D	0.4821	0.3838	−0.0135	0.040*	
C9D	0.50870 (7)	0.23649 (12)	−0.01824 (12)	0.0335 (3)	
H9D	0.5499	0.2495	−0.0241	0.040*	
C10D	0.48740 (7)	0.13777 (12)	−0.01523 (13)	0.0354 (4)	
H10D	0.5152	0.0835	−0.0171	0.042*	
C11D	0.34799 (6)	−0.01830 (11)	0.04801 (12)	0.0316 (3)	
C12D	0.14866 (7)	0.07449 (13)	0.00158 (15)	0.0408 (4)	

### Atomic displacement parameters (Å^2^)

**Table:**

	*U*^11^	*U*^22^	*U*^33^	*U*^12^	*U*^13^	*U*^23^
N1	0.0254 (6)	0.0296 (6)	0.0288 (7)	0.0007 (5)	0.0007 (5)	0.0007 (5)
N2	0.0283 (6)	0.0308 (6)	0.0447 (8)	−0.0002 (5)	0.0013 (6)	0.0033 (6)
N3	0.0288 (6)	0.0296 (7)	0.0544 (9)	0.0026 (5)	0.0009 (6)	−0.0007 (6)
N4	0.0313 (7)	0.0405 (8)	0.0889 (14)	0.0008 (6)	0.0094 (8)	0.0011 (8)
C1	0.0251 (6)	0.0272 (7)	0.0252 (7)	0.0023 (5)	0.0006 (5)	0.0008 (6)
C2	0.0249 (7)	0.0305 (7)	0.0338 (8)	0.0020 (5)	0.0014 (6)	0.0012 (6)
C3	0.0298 (7)	0.0295 (7)	0.0421 (9)	0.0059 (6)	0.0019 (7)	0.0009 (6)
C4	0.0312 (7)	0.0253 (7)	0.0399 (9)	0.0008 (6)	0.0013 (6)	0.0000 (6)
C5	0.0284 (7)	0.0267 (7)	0.0259 (8)	0.0010 (5)	0.0000 (6)	0.0013 (6)
C6	0.0284 (7)	0.0266 (7)	0.0303 (8)	−0.0017 (5)	0.0010 (6)	−0.0022 (6)
C7	0.0327 (8)	0.0286 (7)	0.0381 (9)	0.0006 (6)	0.0014 (7)	0.0009 (6)
C8	0.0378 (8)	0.0307 (8)	0.0408 (10)	−0.0074 (6)	0.0056 (7)	−0.0003 (7)
C9	0.0280 (7)	0.0379 (8)	0.0425 (10)	−0.0050 (6)	0.0035 (7)	−0.0059 (7)
C10	0.0275 (7)	0.0352 (8)	0.0495 (11)	−0.0003 (6)	−0.0013 (7)	0.0013 (7)
C11	0.0247 (6)	0.0269 (7)	0.0328 (8)	−0.0013 (5)	−0.0003 (6)	0.0023 (6)
C12	0.0301 (8)	0.0306 (8)	0.0515 (11)	0.0036 (6)	0.0035 (7)	0.0012 (7)
N1B	0.0271 (6)	0.0304 (6)	0.0407 (8)	0.0020 (5)	0.0013 (5)	0.0014 (5)
N2B	0.0306 (7)	0.0301 (7)	0.0675 (11)	−0.0003 (5)	0.0044 (7)	0.0042 (7)
N3B	0.0304 (7)	0.0296 (7)	0.1174 (17)	0.0005 (6)	−0.0046 (8)	0.0034 (8)
N4B	0.0302 (7)	0.0396 (8)	0.0866 (13)	0.0031 (6)	0.0030 (8)	−0.0004 (8)
C1B	0.0276 (7)	0.0283 (7)	0.0346 (9)	0.0028 (6)	0.0008 (6)	0.0017 (6)
C2B	0.0276 (7)	0.0343 (8)	0.0485 (10)	0.0038 (6)	0.0022 (7)	0.0033 (7)
C3B	0.0324 (8)	0.0307 (8)	0.0859 (16)	0.0071 (7)	0.0016 (9)	0.0053 (9)
C4B	0.0365 (9)	0.0276 (8)	0.0789 (15)	0.0030 (7)	0.0010 (9)	0.0052 (8)
C5B	0.0309 (7)	0.0281 (7)	0.0403 (9)	0.0005 (6)	0.0010 (7)	0.0028 (6)
C6B	0.0312 (7)	0.0296 (7)	0.0367 (9)	−0.0006 (6)	0.0026 (6)	0.0033 (6)
C7B	0.0369 (8)	0.0294 (7)	0.0426 (10)	−0.0004 (6)	0.0009 (7)	0.0019 (7)
C8B	0.0403 (9)	0.0318 (8)	0.0421 (10)	−0.0063 (7)	0.0026 (7)	0.0005 (7)
C9B	0.0324 (8)	0.0414 (9)	0.0381 (9)	−0.0054 (7)	0.0048 (7)	0.0006 (7)
C10B	0.0311 (8)	0.0365 (9)	0.0589 (12)	0.0004 (6)	0.0051 (8)	0.0026 (8)
C11B	0.0258 (7)	0.0299 (8)	0.0628 (12)	−0.0014 (6)	−0.0004 (7)	−0.0010 (7)
C12B	0.0326 (8)	0.0313 (8)	0.0584 (12)	0.0066 (6)	0.0022 (8)	0.0019 (7)
N1C	0.0265 (6)	0.0347 (7)	0.0373 (8)	−0.0035 (5)	0.0023 (5)	0.0045 (6)
N2C	0.0299 (7)	0.0340 (8)	0.0930 (14)	−0.0011 (6)	0.0061 (8)	−0.0108 (8)
N3C	0.0309 (7)	0.0359 (8)	0.1179 (17)	−0.0034 (6)	0.0041 (9)	0.0105 (9)
N4C	0.0310 (7)	0.0441 (9)	0.0810 (13)	−0.0046 (6)	0.0042 (7)	0.0110 (8)
C1C	0.0274 (7)	0.0320 (7)	0.0327 (8)	−0.0036 (6)	0.0021 (6)	0.0008 (6)
C2C	0.0274 (7)	0.0362 (8)	0.0384 (9)	−0.0044 (6)	0.0022 (6)	0.0024 (7)
C3C	0.0317 (8)	0.0364 (9)	0.0607 (12)	−0.0098 (7)	0.0035 (8)	−0.0051 (8)
C4C	0.0351 (8)	0.0315 (8)	0.0605 (12)	−0.0036 (6)	0.0051 (8)	−0.0065 (8)
C5C	0.0302 (7)	0.0321 (7)	0.0353 (9)	−0.0025 (6)	0.0029 (6)	−0.0029 (6)
C6C	0.0304 (7)	0.0322 (8)	0.0344 (9)	−0.0009 (6)	0.0048 (6)	−0.0023 (6)
C7C	0.0356 (8)	0.0345 (8)	0.0554 (12)	−0.0010 (7)	−0.0009 (8)	−0.0032 (8)
C8C	0.0389 (9)	0.0346 (8)	0.0599 (12)	0.0052 (7)	0.0024 (8)	−0.0014 (8)
C9C	0.0315 (8)	0.0403 (9)	0.0491 (11)	0.0026 (7)	0.0050 (7)	0.0010 (8)
C10C	0.0287 (8)	0.0405 (10)	0.0892 (16)	−0.0020 (7)	0.0051 (9)	−0.0084 (10)
C11C	0.0261 (7)	0.0348 (8)	0.0592 (12)	0.0004 (6)	0.0035 (7)	0.0040 (8)
C12C	0.0310 (8)	0.0367 (8)	0.0512 (11)	−0.0067 (6)	0.0021 (7)	0.0064 (7)
N1D	0.0237 (6)	0.0334 (6)	0.0364 (8)	−0.0002 (5)	0.0019 (5)	0.0001 (5)
N2D	0.0270 (6)	0.0299 (6)	0.0414 (8)	−0.0011 (5)	0.0003 (5)	0.0011 (6)
N3D	0.0279 (6)	0.0343 (7)	0.0671 (11)	0.0000 (5)	0.0006 (7)	0.0067 (7)
N4D	0.0283 (7)	0.0475 (9)	0.0878 (14)	0.0010 (6)	0.0033 (8)	0.0088 (8)
C1D	0.0248 (7)	0.0286 (7)	0.0286 (8)	0.0018 (5)	−0.0001 (6)	−0.0009 (6)
C2D	0.0241 (7)	0.0347 (8)	0.0384 (9)	0.0023 (6)	0.0003 (6)	−0.0026 (7)
C3D	0.0285 (7)	0.0330 (8)	0.0526 (11)	0.0054 (6)	−0.0042 (7)	−0.0016 (7)
C4D	0.0328 (8)	0.0280 (7)	0.0464 (10)	0.0016 (6)	−0.0050 (7)	0.0006 (7)
C5D	0.0268 (7)	0.0298 (7)	0.0276 (8)	−0.0001 (6)	−0.0006 (6)	−0.0030 (6)
C6D	0.0268 (7)	0.0294 (7)	0.0256 (8)	−0.0007 (5)	−0.0011 (6)	−0.0008 (6)
C7D	0.0323 (7)	0.0309 (7)	0.0304 (8)	−0.0016 (6)	0.0010 (6)	−0.0004 (6)
C8D	0.0362 (8)	0.0314 (7)	0.0330 (9)	−0.0073 (6)	0.0007 (6)	0.0010 (6)
C9D	0.0284 (7)	0.0386 (8)	0.0332 (9)	−0.0053 (6)	0.0000 (6)	0.0008 (7)
C10D	0.0260 (7)	0.0361 (8)	0.0438 (10)	−0.0009 (6)	0.0008 (7)	0.0009 (7)
C11D	0.0234 (7)	0.0313 (8)	0.0401 (9)	−0.0036 (6)	0.0011 (6)	0.0007 (6)
C12D	0.0291 (8)	0.0388 (9)	0.0545 (11)	0.0043 (7)	0.0019 (7)	0.0028 (8)

### Geometric parameters (Å, º)

**Table:**

N1—C2	1.3338 (18)	N1C—C2C	1.3290 (19)
N1—C1	1.3410 (18)	N1C—C1C	1.3454 (19)
N2—C6	1.3384 (18)	N2C—C6C	1.333 (2)
N2—C10	1.3392 (19)	N2C—C10C	1.341 (2)
N3—C11	1.1416 (19)	N3C—C11C	1.142 (2)
N4—C12	1.141 (2)	N4C—C12C	1.144 (2)
C1—C5	1.4077 (19)	C1C—C5C	1.409 (2)
C1—C11	1.4540 (19)	C1C—C11C	1.448 (2)
C2—C3	1.392 (2)	C2C—C3C	1.383 (2)
C2—C12	1.449 (2)	C2C—C12C	1.452 (2)
C3—C4	1.373 (2)	C3C—C4C	1.380 (2)
C3—H3	0.9500	C3C—H3C	0.9500
C4—C5	1.393 (2)	C4C—C5C	1.392 (2)
C4—H4	0.9500	C4C—H4C	0.9500
C5—C6	1.486 (2)	C5C—C6C	1.486 (2)
C6—C7	1.394 (2)	C6C—C7C	1.386 (2)
C7—C8	1.384 (2)	C7C—C8C	1.383 (2)
C7—H7	0.9500	C7C—H7C	0.9500
C8—C9	1.376 (2)	C8C—C9C	1.371 (2)
C8—H8	0.9500	C8C—H8C	0.9500
C9—C10	1.381 (2)	C9C—C10C	1.371 (2)
C9—H9	0.9500	C9C—H9C	0.9500
C10—H10	0.9500	C10C—H10C	0.9500
N1B—C2B	1.3301 (19)	N1D—C2D	1.3342 (18)
N1B—C1B	1.3424 (18)	N1D—C1D	1.3437 (18)
N2B—C6B	1.336 (2)	N2D—C6D	1.3403 (19)
N2B—C10B	1.337 (2)	N2D—C10D	1.3424 (19)
N3B—C11B	1.142 (2)	N3D—C11D	1.1443 (19)
N4B—C12B	1.140 (2)	N4D—C12D	1.140 (2)
C1B—C5B	1.405 (2)	C1D—C5D	1.404 (2)
C1B—C11B	1.452 (2)	C1D—C11D	1.454 (2)
C2B—C3B	1.384 (2)	C2D—C3D	1.383 (2)
C2B—C12B	1.451 (2)	C2D—C12D	1.454 (2)
C3B—C4B	1.373 (2)	C3D—C4D	1.378 (2)
C3B—H3B	0.9500	C3D—H3D	0.9500
C4B—C5B	1.393 (2)	C4D—C5D	1.394 (2)
C4B—H4B	0.9500	C4D—H4D	0.9500
C5B—C6B	1.484 (2)	C5D—C6D	1.4879 (19)
C6B—C7B	1.390 (2)	C6D—C7D	1.394 (2)
C7B—C8B	1.382 (2)	C7D—C8D	1.385 (2)
C7B—H7B	0.9500	C7D—H7D	0.9500
C8B—C9B	1.373 (2)	C8D—C9D	1.378 (2)
C8B—H8B	0.9500	C8D—H8D	0.9500
C9B—C10B	1.381 (2)	C9D—C10D	1.386 (2)
C9B—H9B	0.9500	C9D—H9D	0.9500
C10B—H10B	0.9500	C10D—H10D	0.9500
			
C2—N1—C1	116.28 (12)	C2C—N1C—C1C	116.78 (13)
C6—N2—C10	117.29 (13)	C6C—N2C—C10C	117.86 (14)
N1—C1—C5	124.69 (13)	N1C—C1C—C5C	124.31 (13)
N1—C1—C11	112.09 (12)	N1C—C1C—C11C	111.17 (13)
C5—C1—C11	123.20 (12)	C5C—C1C—C11C	124.51 (13)
N1—C2—C3	124.35 (13)	N1C—C2C—C3C	124.24 (14)
N1—C2—C12	115.62 (13)	N1C—C2C—C12C	115.38 (14)
C3—C2—C12	120.02 (13)	C3C—C2C—C12C	120.38 (14)
C4—C3—C2	117.93 (13)	C4C—C3C—C2C	118.04 (15)
C4—C3—H3	121.0	C4C—C3C—H3C	121.0
C2—C3—H3	121.0	C2C—C3C—H3C	121.0
C3—C4—C5	120.52 (14)	C3C—C4C—C5C	120.61 (15)
C3—C4—H4	119.7	C3C—C4C—H4C	119.7
C5—C4—H4	119.7	C5C—C4C—H4C	119.7
C4—C5—C1	116.21 (13)	C4C—C5C—C1C	115.99 (14)
C4—C5—C6	121.87 (13)	C4C—C5C—C6C	121.63 (14)
C1—C5—C6	121.92 (12)	C1C—C5C—C6C	122.35 (13)
N2—C6—C7	122.78 (13)	N2C—C6C—C7C	122.05 (14)
N2—C6—C5	116.04 (13)	N2C—C6C—C5C	116.17 (14)
C7—C6—C5	121.18 (13)	C7C—C6C—C5C	121.75 (14)
C8—C7—C6	118.58 (14)	C8C—C7C—C6C	118.90 (15)
C8—C7—H7	120.7	C8C—C7C—H7C	120.6
C6—C7—H7	120.7	C6C—C7C—H7C	120.6
C9—C8—C7	119.12 (15)	C9C—C8C—C7C	119.33 (16)
C9—C8—H8	120.4	C9C—C8C—H8C	120.3
C7—C8—H8	120.4	C7C—C8C—H8C	120.3
C8—C9—C10	118.45 (14)	C8C—C9C—C10C	118.14 (15)
C8—C9—H9	120.8	C8C—C9C—H9C	120.9
C10—C9—H9	120.8	C10C—C9C—H9C	120.9
N2—C10—C9	123.76 (15)	N2C—C10C—C9C	123.71 (16)
N2—C10—H10	118.1	N2C—C10C—H10C	118.1
C9—C10—H10	118.1	C9C—C10C—H10C	118.1
N3—C11—C1	172.44 (15)	N3C—C11C—C1C	170.50 (17)
N4—C12—C2	178.2 (2)	N4C—C12C—C2C	178.75 (19)
C2B—N1B—C1B	116.69 (13)	C2D—N1D—C1D	116.55 (13)
C6B—N2B—C10B	118.18 (14)	C6D—N2D—C10D	117.67 (13)
N1B—C1B—C5B	124.70 (13)	N1D—C1D—C5D	124.58 (13)
N1B—C1B—C11B	111.34 (13)	N1D—C1D—C11D	111.24 (12)
C5B—C1B—C11B	123.95 (13)	C5D—C1D—C11D	124.17 (12)
N1B—C2B—C3B	124.03 (14)	N1D—C2D—C3D	124.30 (13)
N1B—C2B—C12B	116.38 (14)	N1D—C2D—C12D	115.07 (14)
C3B—C2B—C12B	119.59 (14)	C3D—C2D—C12D	120.63 (14)
C4B—C3B—C2B	118.00 (15)	C4D—C3D—C2D	117.84 (14)
C4B—C3B—H3B	121.0	C4D—C3D—H3D	121.1
C2B—C3B—H3B	121.0	C2D—C3D—H3D	121.1
C3B—C4B—C5B	120.98 (15)	C3D—C4D—C5D	120.75 (14)
C3B—C4B—H4B	119.5	C3D—C4D—H4D	119.6
C5B—C4B—H4B	119.5	C5D—C4D—H4D	119.6
C4B—C5B—C1B	115.57 (14)	C4D—C5D—C1D	115.97 (13)
C4B—C5B—C6B	121.77 (14)	C4D—C5D—C6D	121.10 (13)
C1B—C5B—C6B	122.64 (13)	C1D—C5D—C6D	122.91 (13)
N2B—C6B—C7B	121.87 (14)	N2D—C6D—C7D	122.40 (13)
N2B—C6B—C5B	115.92 (13)	N2D—C6D—C5D	116.34 (12)
C7B—C6B—C5B	122.20 (14)	C7D—C6D—C5D	121.25 (13)
C8B—C7B—C6B	119.04 (15)	C8D—C7D—C6D	118.96 (14)
C8B—C7B—H7B	120.5	C8D—C7D—H7D	120.5
C6B—C7B—H7B	120.5	C6D—C7D—H7D	120.5
C9B—C8B—C7B	119.30 (15)	C9D—C8D—C7D	119.06 (14)
C9B—C8B—H8B	120.3	C9D—C8D—H8D	120.5
C7B—C8B—H8B	120.3	C7D—C8D—H8D	120.5
C8B—C9B—C10B	118.17 (15)	C8D—C9D—C10D	118.44 (14)
C8B—C9B—H9B	120.9	C8D—C9D—H9D	120.8
C10B—C9B—H9B	120.9	C10D—C9D—H9D	120.8
N2B—C10B—C9B	123.44 (15)	N2D—C10D—C9D	123.43 (14)
N2B—C10B—H10B	118.3	N2D—C10D—H10D	118.3
C9B—C10B—H10B	118.3	C9D—C10D—H10D	118.3
N3B—C11B—C1B	170.85 (17)	N3D—C11D—C1D	170.90 (15)
N4B—C12B—C2B	177.79 (17)	N4D—C12D—C2D	179.5 (2)
			
C2—N1—C1—C5	−0.7 (2)	C2C—N1C—C1C—C5C	1.1 (2)
C2—N1—C1—C11	−178.87 (13)	C2C—N1C—C1C—C11C	−178.13 (15)
C1—N1—C2—C3	1.5 (2)	C1C—N1C—C2C—C3C	0.6 (3)
C1—N1—C2—C12	−177.60 (14)	C1C—N1C—C2C—C12C	−178.59 (14)
N1—C2—C3—C4	−1.2 (3)	N1C—C2C—C3C—C4C	−1.6 (3)
C12—C2—C3—C4	177.83 (15)	C12C—C2C—C3C—C4C	177.56 (17)
C2—C3—C4—C5	0.2 (2)	C2C—C3C—C4C—C5C	0.9 (3)
C3—C4—C5—C1	0.5 (2)	C3C—C4C—C5C—C1C	0.5 (3)
C3—C4—C5—C6	−179.31 (15)	C3C—C4C—C5C—C6C	−177.69 (17)
N1—C1—C5—C4	−0.2 (2)	N1C—C1C—C5C—C4C	−1.6 (2)
C11—C1—C5—C4	177.73 (14)	C11C—C1C—C5C—C4C	177.49 (17)
N1—C1—C5—C6	179.60 (14)	N1C—C1C—C5C—C6C	176.61 (15)
C11—C1—C5—C6	−2.5 (2)	C11C—C1C—C5C—C6C	−4.3 (3)
C10—N2—C6—C7	−1.6 (2)	C10C—N2C—C6C—C7C	−0.4 (3)
C10—N2—C6—C5	178.21 (14)	C10C—N2C—C6C—C5C	−178.60 (18)
C4—C5—C6—N2	154.51 (15)	C4C—C5C—C6C—N2C	167.51 (17)
C1—C5—C6—N2	−25.3 (2)	C1C—C5C—C6C—N2C	−10.6 (2)
C4—C5—C6—C7	−25.7 (2)	C4C—C5C—C6C—C7C	−10.7 (3)
C1—C5—C6—C7	154.52 (15)	C1C—C5C—C6C—C7C	171.18 (17)
N2—C6—C7—C8	0.6 (2)	N2C—C6C—C7C—C8C	−0.3 (3)
C5—C6—C7—C8	−179.14 (14)	C5C—C6C—C7C—C8C	177.86 (17)
C6—C7—C8—C9	0.9 (2)	C6C—C7C—C8C—C9C	0.4 (3)
C7—C8—C9—C10	−1.3 (3)	C7C—C8C—C9C—C10C	0.1 (3)
C6—N2—C10—C9	1.1 (3)	C6C—N2C—C10C—C9C	1.0 (3)
C8—C9—C10—N2	0.4 (3)	C8C—C9C—C10C—N2C	−0.8 (3)
C2B—N1B—C1B—C5B	0.3 (2)	C2D—N1D—C1D—C5D	−0.5 (2)
C2B—N1B—C1B—C11B	−178.58 (15)	C2D—N1D—C1D—C11D	178.94 (14)
C1B—N1B—C2B—C3B	1.1 (3)	C1D—N1D—C2D—C3D	0.0 (2)
C1B—N1B—C2B—C12B	−178.75 (15)	C1D—N1D—C2D—C12D	−179.83 (15)
N1B—C2B—C3B—C4B	−1.2 (3)	N1D—C2D—C3D—C4D	0.3 (3)
C12B—C2B—C3B—C4B	178.61 (19)	C12D—C2D—C3D—C4D	−179.86 (16)
C2B—C3B—C4B—C5B	−0.1 (3)	C2D—C3D—C4D—C5D	−0.2 (3)
C3B—C4B—C5B—C1B	1.3 (3)	C3D—C4D—C5D—C1D	−0.2 (2)
C3B—C4B—C5B—C6B	−177.33 (18)	C3D—C4D—C5D—C6D	178.30 (15)
N1B—C1B—C5B—C4B	−1.5 (3)	N1D—C1D—C5D—C4D	0.6 (2)
C11B—C1B—C5B—C4B	177.32 (18)	C11D—C1D—C5D—C4D	−178.73 (15)
N1B—C1B—C5B—C6B	177.12 (15)	N1D—C1D—C5D—C6D	−177.91 (14)
C11B—C1B—C5B—C6B	−4.1 (3)	C11D—C1D—C5D—C6D	2.8 (2)
C10B—N2B—C6B—C7B	−0.1 (3)	C10D—N2D—C6D—C7D	−0.4 (2)
C10B—N2B—C6B—C5B	−179.15 (16)	C10D—N2D—C6D—C5D	178.54 (14)
C4B—C5B—C6B—N2B	174.30 (17)	C4D—C5D—C6D—N2D	−162.62 (15)
C1B—C5B—C6B—N2B	−4.2 (2)	C1D—C5D—C6D—N2D	15.8 (2)
C4B—C5B—C6B—C7B	−4.7 (3)	C4D—C5D—C6D—C7D	16.4 (2)
C1B—C5B—C6B—C7B	176.74 (16)	C1D—C5D—C6D—C7D	−165.21 (15)
N2B—C6B—C7B—C8B	−0.7 (3)	N2D—C6D—C7D—C8D	1.5 (2)
C5B—C6B—C7B—C8B	178.25 (16)	C5D—C6D—C7D—C8D	−177.44 (14)
C6B—C7B—C8B—C9B	0.9 (3)	C6D—C7D—C8D—C9D	−0.8 (2)
C7B—C8B—C9B—C10B	−0.3 (3)	C7D—C8D—C9D—C10D	−0.8 (2)
C6B—N2B—C10B—C9B	0.8 (3)	C6D—N2D—C10D—C9D	−1.3 (2)
C8B—C9B—C10B—N2B	−0.6 (3)	C8D—C9D—C10D—N2D	1.9 (3)

### Hydrogen-bond geometry (Å, º)

**Table:**

*D*—H···*A*	*D*—H	H···*A*	*D*···*A*	*D*—H···*A*
C3—H3···N3^i^	0.95	2.42	3.343 (2)	164
C3*B*—H3*B*···N3*B*^ii^	0.95	2.34	3.281 (2)	169
C10*B*—H10*B*···N3	0.95	2.57	3.269 (2)	130
C3*C*—H3*C*···N3*D*^iii^	0.95	2.46	3.379 (2)	164
C3*D*—H3*D*···N3*C*	0.95	2.56	3.397 (2)	145

Symmetry codes: (i) −*x*+1, *y*+1/2, −*z*+1/2; (ii) −*x*+2, *y*−1/2, −*z*+1/2; (iii) *x*, *y*+1, *z*.
